# Noise-resilient spatial–channel refinement for robust remote photoplethysmography

**DOI:** 10.3389/fbioe.2026.1819324

**Published:** 2026-05-07

**Authors:** Xianwei Zhang, Feng Qiao, Qiaochu Zang, Zhe Chen, Xiaoting Xie, Siwen Zhao, Sen Lin, Yujun Li

**Affiliations:** School of Information Science and Engineering, Shandong University, Qingdao, China

**Keywords:** deep learning, heart rate estimation, multi-scale frame difference fusion, remote photoplethysmography, spatial and channel attention

## Abstract

**Introduction:**

Remote photoplethysmography (rPPG) enables non-contact heart rate measurement from facial videos with growing health monitoring applications. Despite advances in deep learning methods, most existing approaches lack task-specific feature refinement across spatial and channel dimensions, which can dilute pulsatile information.

**Methods:**

We propose two key contributions to address this challenge. First, a Multi-scale Difference Fusion Stem (MDFS) captures temporal dynamics through learnable fusion of multi-scale frame differences, providing noise-resilient representations. Second, a Spatial-Channel Optimized Pulse Enhancement (SCOPE) module performs adaptive spatial-channel refinement with gated fusion to amplify pulse-related features while suppressing interference.

**Results:**

Comprehensive experiments on PURE, UBFC-rPPG, and MMPD datasets demonstrate state-of-the-art performance in both intra-dataset and cross-dataset evaluations.

**Discussion:**

These results indicate that explicitly constructing multi-scale temporal cues and performing controlled spatial-channel refinement are effective for robust rPPG estimation under low-SNR conditions.

## 1 Introduction

Heart rate (HR) is a fundamental biomarker of cardiovascular health, making non-contact, real-time HR monitoring an important research topic McDuff (2023); Al-Naji et al. (2017). Conventional photoplethysmography (PPG) measures blood-volume pulse waveforms through optical sensors in direct skin contact, enabling the extraction of physiological parameters such as blood pressure Chowdhury et al. (2020) and HR Shuzan et al. (2021). However, this contact requirement limits PPG applicability in scenarios where physical contact is impractical or undesirable Chen et al. (2024). Remote photoplethysmography (rPPG) addresses this limitation by detecting subtle cardiac-induced skin color variations, enabling fully contactless HR estimation Boccignone et al. (2022).

Early rPPG methods relied on handcrafted signal-processing techniques Verkruysse et al. (2008); Poh et al. (2010); Lewandowska and Nowak (2012); De Haan and Jeanne (2013); Wang et al. (2016), which were highly susceptible to motion artifacts and illumination changes Sun and Thakor (2015); Zhan et al. (2020). The advent of deep learning has brought significant improvements, with convolutional networks and transformer architectures achieving substantially enhanced robustness and accuracy Chen and McDuff (2018); Yu et al. (2022). However, most existing designs still lack task-specific feature refinement across spatial regions and channel responses, which can dilute pulsatile information and impair generalization under cross-scenario and cross-dataset settings Woo et al. (2018). This limitation stems from the unique characteristics of rPPG signals—subtle amplitude variations, high susceptibility to interference, and inherent periodicity—which demand specialized mechanisms beyond general-purpose attention modules.

To address the above challenges, we propose a unified rPPG framework built upon two complementary designs: Spatial-Channel Optimized Pulse Enhancement (SCOPE) and Multi-scale Difference Fusion Stem (MDFS). Specifically, SCOPE performs rPPG-oriented spatial–channel refinement to emphasize pulse-responsive patterns while suppressing non-pulsatile interference, and employs a gated fusion strategy to enhance discriminative features without over-amplifying noise. Complementary to SCOPE, MDFS enriches the input representation by explicitly modeling multi-scale temporal differences and adaptively fusing them with learnable weights, enabling robust extraction of pulsatile dynamics across different temporal frequencies.

We evaluate the proposed framework on multiple benchmark datasets under both intra-dataset and cross-dataset settings, including UBFC-rPPG Bobbia et al. (2019), PURE Stricker et al. (2014), and MMPD Tang et al. (2023). Experimental results show that integrating MDFS and SCOPE consistently improves performance over representative baselines across most metrics, demonstrating strong robustness and generalization under low-SNR conditions.

The main contributions of this work are summarized as follows.

- We propose MDFS, which captures multi-scale temporal variations via learnable fusion of frame differences to build noise-resilient input representations.
- We design SCOPE, an rPPG-specific spatial–channel refinement module with gated enhancement to amplify pulse-related features while suppressing noise.
- Extensive experiments on UBFC-rPPG, PURE, and MMPD demonstrate that the proposed method achieves state-of-the-art performance with strong cross-dataset generalization.

## 2 Related work

### 2.1 rPPG signal measurement methods

Measurement methods for rPPG signals can be broadly divided into two categories: traditional rPPG measurement methods and deep-learning-based approaches. The earliest traditional method is the GREEN algorithm proposed by Verkruysse et al. Verkruysse et al. (2008), and subsequent works develop additional signal-processing techniques, including blind source separation (BSS) methods such as independent component analysis (ICA) Poh et al. (2010), principal component analysis (PCA) Lewandowska and Nowak (2012), chrominance-based methods (CHROM) De Haan and Jeanne (2013), and the plane-orthogonal-to-skin (POS) projection Wang et al. (2016). As summarized above, traditional rPPG algorithms primarily process the characteristics of the BVP signal, but they are mostly developed under relatively controlled conditions and are therefore vulnerable to noise in real-world scenarios Sun and Thakor (2015); Zhan et al. (2020); Feng et al. (2014).

With the rise of deep learning, rPPG estimation has made notable progress. DeepPhys Chen and McDuff (2018) first introduced neural networks into rPPG signal modeling, while PhysNet Yu et al. (2019) adopted 3D convolutional kernels to capture spatiotemporal features. Subsequently, Transformer-based architectures have further improved robustness and generalization. For example, PhysFormer Yu et al. (2022) used temporal differences to guide global attention and strengthen quasi-periodic features, and EfficientPhys Liu et al. (2023a) reduced computational complexity and improved cross-dataset generalization through lightweight design and multi-scale feature extraction. Subsequent works explored specialized designs, such as Spiking-PhysFormer Liu et al. (2025), which incorporated spiking neural networks for lower power consumption, and LGI-rPPG-Net Chowdhury et al. (2024), which employed a shallow encoder–decoder structure for efficient recovery. More recent studies have addressed low-resolution scenarios, including anti-aliasing convolution with spatiotemporal attention Xue et al. (2025) and adversarial learning frameworks for signal recovery Zhai et al. (2025). RhythmFormer Zou et al. (2025) further introduced a periodic sparse attention mechanism to reduce redundant computation and bias in self-attention.

Despite these advances, most rPPG methods still rely on raw frames or fixed single-scale temporal differences, which are fragile under motion and illumination noise. Moreover, generic attention designs lack rPPG-specific, controllable spatial–channel refinement, motivating a model that explicitly constructs noise-resilient temporal cues and task-aware feature enhancement.

### 2.2 Spatial–channel attention mechanism

Most existing networks design spatial and channel attention separately. For example, Woo et al. Woo et al. (2018) in CBAM sequentially infer channel and spatial attention, but without explicit joint optimization. In the rPPG domain, DeepPhys Chen and McDuff (2018) applies spatial attention to emphasize facial regions reflecting pulsatile signals, but it only focuses on the spatial dimension without joint channel optimization. TS-CAN Liu et al. (2020) introduces a spatial–temporal attention mechanism, but its design mainly focuses on the spatial and temporal dimensions, with insufficient modeling of channel-wise selectivity. Although recent studies attempt to improve attention design, RhythmFormer Zou et al. (2025) leverages periodic sparse attention to exploit task-specific priors for enhanced modeling, and Spore Zhang et al. (2025) enhances feature discriminability and generalization through spatio-temporal co-perception and disentangled representation. However, dynamic co-optimization of spatial and channel attention remains underexplored, which limits performance in low signal-to-noise ratio scenarios.

Different from previous approaches that independently model spatial or channel attention at the global level, we explicitly integrate their collaborative relationship into the attention mechanism. The proposed spatial–channel attention adaptively highlights heartbeat-related channels and focuses on pulse-sensitive regions such as cheeks and forehead, while suppressing irrelevant features. This design improves the capture of low-SNR pulsatile waveforms and enables more fine-grained feature modeling.

## 3 Methods

In Section 3.1, we present the overall framework of the system, followed by the introduction of MDFS in Section 3.2, SCOPE in Section 3.3.

### 3.1 Overall framework

**FIGURE 1:**
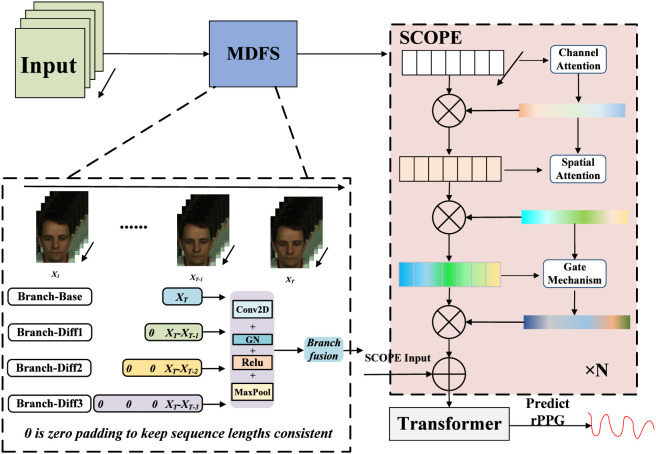
Overall framework of the proposed rPPG extraction system. MDFS performs multi-scale inter-frame difference modeling, and the fused features are fed into a transformer-based temporal backbone with periodic sparse attention, where SCOPE blocks refine spatial–channel representations for robust rPPG waveform prediction.

The proposed framework is shown in Figure 1. It comprises three components: MDFS for multi-scale temporal cue construction, a transformer-based temporal backbone for long-range dependency modeling, and SCOPE modules for spatial–channel refinement. MDFS first enhances input representations by exposing subtle pulsatile dynamics, after which temporal modeling and SCOPE-based refinement are performed in an interleaved manner to suppress noise and strengthen pulse-related features. This design enables robust rPPG waveform prediction under low-SNR conditions.

### 3.2 MDFS

rPPG estimation is inherently challenged by low-SNR facial signals that are easily corrupted by motion and illumination disturbances Sun and Thakor (2015); Zhan et al. (2020); Feng et al. (2014). To suppress static components and expose subtle temporal variations, we adopt an input enhancement scheme based on frame differences Ren et al. (2022), which has also been used in recent transformer-based rPPG pipelines Yu et al. (2022); Niu et al. (2019); Zhang et al. (2025). However, a fixed single-scale difference remains fragile under noise and cannot reliably capture pulsatile dynamics with varying temporal characteristics.

To this end, we propose the MDFS. MDFS computes multi-scale frame differences and performs learnable fusion along the difference-scale dimension, enabling adaptive emphasis on the most informative temporal variations for subsequent modeling.

Specifically, given an input video sequence $X\in R^{N\times D\times C\times H\times W}$ (where D = 160 frames per clip), we denote by $X^{\left(n,t\right)}\in R^{C\times H\times W}$ the t -th frame of the n -th sample, where n∈{1,…,N} indexes samples in the batch, t∈{1,…,D} indexes frame positions within each clip, C is the number of channels, and (H,W) is the spatial resolution. For each temporal scale s∈{1,2,3} , the s -frame difference is computed as shown in Equation 1:

$$\Delta_{s}^{\left(n,t\right)}=\left\{\begin{array}{cc} X^{\left(n,t\right)}-X^{\left(n,t-s\right)}, & t\geq s,\\ 0, & t<s, \end{array}\right.$$ (1)

where $0\in R^{C\times H\times W}$ denotes zero padding for the first s frames, ensuring temporal alignment across all four streams. In this way, we obtain three difference sequences $\left\{\Delta_{1},\Delta_{2},\Delta_{3}\right\}$ , which, together with the original frames X , form four parallel input streams.

Each stream is then processed by a lightweight convolutional branch that performs spatial downsampling and channel projection. Let ${\text{Branch}}_{0}\left(\cdot \right)$ and ${\text{Branch}}_{s}\left(\cdot \right)$ denote the convolutional blocks for the original and s -frame difference streams, respectively. For the (n,t) -th frame, their outputs are defined in Equations 2,3, with the feature dimensions specified in Equation 4:

$$f_{0}^{\left(n,t\right)}={\text{Branch}}_{0}\left(X^{\left(n,t\right)}\right),$$ (2)

$$f_{s}^{\left(n,t\right)}={\text{Branch}}_{s}\left(\Delta_{s}^{\left(n,t\right)}\right),\;s=1,2,3,$$ (3)

where

$$f_{i}^{\left(n,t\right)}\in R^{C_{\text{mid}}\times \frac{H}{4}\times \frac{W}{4}},\;i=0,1,2,3,$$ (4)

and $C_{\text{mid}}$ is the intermediate channel dimension shared by all branches.

To improve robustness under noisy conditions, each branch output is further regularized by a Dropout2d layer, as shown in Equation 5:

$${\tilde{f}}_{i}^{\left(n,t\right)}=\text{Dropout}2\text{d}\left(f_{i}^{\left(n,t\right)}\right),\;i=0,1,2,3.$$ (5)

We then introduce learnable parameters $\left\{\partial_{0},\partial_{1},\partial_{2},\partial_{3}\right\}$ to define Softmax weights over the four streams, as formulated in Equation 6:

$$w_{i}=\frac{e^{\partial_{i}}}{\sum_{j=0}^{3}e^{\partial_{j}}},\;\overset{3}{\underset{i=0}{\sum }}w_{i}=1,$$ (6)

and perform weighted fusion according to Equation 7:

$${\hat{f}}^{\left(n,t\right)}=\overset{3}{\underset{i=0}{\sum }}w_{i}\cdot {\tilde{f}}_{i}^{\left(n,t\right)},\;{\hat{f}}^{\left(n,t\right)}\in R^{C_{\text{mid}}\times \frac{H}{4}\times \frac{W}{4}}.$$ (7)

Finally, a ReLU activation is applied to obtain the MDFS output, as shown in Equation 8:

$$X_{\text{MDFS}}^{\left(n,t\right)}=\text{ReLU}\left({\hat{f}}^{\left(n,t\right)}\right).$$ (8)

In summary, MDFS provides a multi-scale temporal representation that is more robust to noise than single-scale differencing, facilitating subsequent temporal modeling with interleaved SCOPE refinement.

### 3.3 SCOPE

rPPG features are inherently low-SNR, easily corrupted by motion and illumination disturbances, and often exhibit strong spatial heterogeneity across facial regions. Motivated by three rPPG-specific challenges, namely, spatially non-uniform pulse distribution across facial regions, the need to model distributed pulse-bearing areas, and the risk of amplifying non-pulsatile noise under low-SNR conditions, we design the SCOPE module as an rPPG-oriented feature refinement block. Rather than introducing a new primitive attention operator, SCOPE performs task-specific refinement by combining spatial-aware channel reweighting, large-receptive-field spatial enhancement, and adaptive gated residual fusion. This design aims to emphasize pulse-responsive facial patterns while suppressing non-pulsatile interference before temporal modeling.

SCOPE consists of three components: channel attention, spatial attention, and an adaptive gated residual fusion module. By integrating these three elements, SCOPE aims to extract pulsatile information from frame-level feature maps, attenuate background interference, and provide cleaner and more discriminative inputs for subsequent temporal modeling. Formally, the input to SCOPE is a 4D tensor $X\in R^{B\times C\times H_{m}\times W_{m}}$ , where B=N×D is the product of batch size N and number of frames D , and $\left(H_{m},W_{m}\right)$ is the spatial resolution after MDFS downsampling ( $H_{m}=H/4$ , $W_{m}=W/4$ ).

#### 3.3.1 Channel sttention submodule

Because the reflection intensity of pulse signals varies across channels, the channel attention submodule learns to reweight channels according to their relevance to blood-flow fluctuations. Concretely, we first reshape X into $X_{\text{perm}}\in R^{B\times \left(H_{m}W_{m}\right)\times C}$ and apply a lightweight two-layer MLP to each spatial location, as shown in Equation 9:

$$A_{\text{chan}}=\sigma \left(\text{MLP}\left(X_{\text{perm}}\right)\right),$$ (9)

where MLP(⋅) compresses the channel dimension to C/r and then restores it to C , and σ(⋅) denotes the Sigmoid function.

After reshaping $A_{\text{chan}}$ back to $R^{B\times C\times H_{m}\times W_{m}}$ , channel-refined features are obtained as shown in Equation 10:

$$X_{\text{chan}}=X⊙A_{\text{chan}},$$ (10)

where ⊙ denotes element-wise multiplication. This spatial-aware formulation is designed for rPPG’s spatial heterogeneity, where pulse signal strength varies significantly across facial regions. Unlike global pooling-based channel attention that assigns one shared channel weight to the entire feature map, this design allows different facial locations to receive different channel responses, which is more suitable for preserving region-specific pulsatile information.

#### 3.3.2 Spatial attention submodule

Although channel attention amplifies pulse-related channels, spatial localization is still required, since regions such as the forehead and cheeks often exhibit more pronounced pulsatile variations. To enlarge the receptive field and enhance robustness, we apply two consecutive 7×7 convolutions with batch normalization and ReLU activation to $X_{\text{chan}}$ , generating a spatial attention map $A_{\text{spa}}\in R^{B\times C\times H_{m}\times W_{m}}$ . The spatially attended features are then computed as shown in Equation 11:

$$X_{\text{spa}}=X_{\text{chan}}⊙A_{\text{spa}},$$ (11)

which retains pixel regions that are highly related to pulsatile changes in the spatial plane while suppressing background interference. The dual 7×7 convolutions provide larger receptive fields to capture spatially distributed pulsatile patterns that often span across forehead and cheek regions.

Different from generic spatial attention that is constructed from compressed channel statistics, our spatial refinement is performed on channel-refined features and preserves richer channel-dependent spatial responses, which is beneficial for highlighting distributed pulse-bearing facial regions. This large-receptive-field refinement is motivated by the fact that pulse-bearing regions, such as the forehead and cheeks, are spatially distributed and require broader contextual modeling.

#### 3.3.3 Gating mechanism and output fusion

Directly using $X_{\text{spa}}$ may lead to excessive enhancement under severe noise. To control enhancement strength, we introduce an adaptive gating mechanism that predicts channel-wise fusion coefficients from global context. Specifically, a global average pooling followed by two 1×1 convolutions with ReLU and Sigmoid activations produces a gating map $g\in R^{B\times C\times 1\times 1}$ . The final output of SCOPE is then given by Equation 12:

$$\hat{X}=g⊙X_{\text{spa}}+X.$$ (12)

This gated residual fusion preserves the original information path while adaptively modulating the refinement magnitude, reducing the risk of noise amplification. Such adaptive control is particularly important for rPPG, where the target physiological signal is subtle and direct enhancement may also amplify non-pulsatile disturbances caused by motion or illumination variation.

Overall, SCOPE provides controlled spatial–channel refinement at the frame level, and the adaptive gating mechanism further regulates the enhancement strength and facilitates robust feature fusion, producing cleaner and more discriminative representations for subsequent temporal modeling and improving robustness under low-SNR conditions.

## 4 Experiments and analyses

In this section, we evaluate our method on multiple rPPG benchmarks under both intra-dataset and cross-dataset settings to assess performance under matched and unseen conditions, and conduct ablation studies to analyze the contribution of each component.

### 4.1 Dataset and experimental settings

#### 4.1.1 Dataset

We evaluate the proposed method on three publicly available datasets: UBFC-rPPG Bobbia et al. (2019), PURE Stricker et al. (2014), and MMPD Tang et al. (2023).

1. UBFC-rPPG Bobbia et al. (2019): CThis dataset consists of 42 facial videos recorded from subjects in a stationary position. The videos are captured with a resolution of 640 × 480 pixels and a frame rate of 30 fps.
2. PURE Stricker et al. (2014): This dataset contains 60 1-min videos from 10 participants across six different activity scenarios, captured under natural lighting conditions with a video resolution of 640×480 and a frame rate of 30 fps.
3. MMPD Tang et al. (2023): Collected by Tsinghua University, this mobile-device video dataset includes 33 participants and 11 h of recordings, covering various skin tones, motions, and lighting conditions. In this study, we use the compressed version, mini-MMPD. The dataset provides both MMPD and mini-MMPD versions, and we adopt mini-MMPD for our experiments.

#### 4.1.2 Experimental settings

In this study, we implement the evaluation platform based on PyTorch and the open-source rPPG toolbox Liu et al. (2023b), ensuring that all methods are implemented within the same codebase for fair comparison. Experiments are conducted on an RTX 3090 GPU using five evaluation metrics: mean absolute error (MAE), root mean square error (RMSE), mean absolute percentage error (MAPE), Pearson correlation coefficient (ρ) , and signal-to-noise ratio (SNR). A unified learning rate of 9e-3 is adopted, with the AdamW optimizer and a batch size of 4. For each video segment, the facial region is cropped and uniformly resized to 128 × 128 pixels to serve as the network input.

We adopt the hybrid time–frequency loss from RhythmFormer Zou et al. (2025) for fair comparison. The total loss is defined as $L_{\text{total}}=\partial •L_{\text{time}}+L_{\text{freq}}$ , where ∂ is set to 0.2. $L_{\text{time}}$ is the negative Pearson correlation coefficient (Equation 13), and $L_{\text{time}}$ is a cross-entropy loss on the spectral-peak distribution (Equation 14).

$$L_{\text{time}}=1-\frac{\sum_{i=1}^{N}\left(s_{i}-\bar{s}\right)\left({\hat{s}}_{i}-\bar{\hat{s}}\right)}{\sqrt{\sum_{i=1}^{N}{\left(s_{i}-\bar{s}\right)}^{2}}\sqrt{\sum_{i=1}^{N}{\left({\hat{s}}_{i}-\bar{\hat{s}}\right)}^{2}}}$$ (13)

$$L_{\text{freq}}=CE\left(\arg\max PSD\left(s\right),PSD\left(\hat{s}\right)\right)$$ (14)

where s denotes the ground truth BVP wave, and $\hat{s}$ denotes the BVP wave predicted by the model.

### 4.2 Intra-dataset evaluation

To evaluate the proposed method under intra-dataset settings, where models are trained and tested on the same dataset, we conduct experiments on PURE Stricker et al. (2014), UBFC-rPPG Bobbia et al. (2019), and MMPD Tang et al. (2023). We follow standard evaluation protocols and summarize the results in Table 1. All methods are trained for 30 epochs, and the final checkpoint is used for evaluation.

**TABLE 1:** Intra-dataset evaluation results on PURE, UBFC-rPPG, and MMPD. The best results are highlighted in **bold**, and the second-best results are underlined. In case of ties, only our method is emphasized for clarity.

Method	PURE			UBFC-rPPG			MMPD		
	MAE ↓	RMSE ↓	ρ↑	MAE ↓	RMSE ↓	ρ↑	MAE ↓	RMSE ↓	ρ↑
HR-CNN Špetlík et al. (2018)	1.84	2.37	0.98	4.9	5.89	0.64	-	-	-
SynRhythm Niu et al. (2018)	2.71	4.86	0.98	5.59	6.82	0.72	-	-	-
DeepPhys Chen and McDuff (2018)	0.83	1.54	0.99	6.27	10.82	0.65	22.27	28.92	−0.03
PhysNet Yu et al. (2019)	2.10	2.60	0.99	2.95	3.67	0.97	4.80	11.80	0.60
Meta-rPPG Lee et al. (2020)	2.52	4.63	0.98	5.97	7.42	0.53	-	-	-
TS-CAN Liu et al. (2020)	2.48	9.01	0.92	1.70	2.72	0.99	9.71	17.22	0.44
CVD Niu et al. (2020)	1.29	2.01	0.98	2.19	3.12	0.99	-	-	-
PulseGAN Song et al. (2021)	2.28	4.29	0.99	1.19	2.10	0.98	-	-	-
Dual-GAN Lu et al. (2021)	0.82	1.31	0.99	0.44	**0.67**	0.99	-	-	-
PhysFormer Yu et al. (2022)	1.10	1.75	0.99	0.50	0.71	0.99	11.99	18.41	0.18
ETA-rPPGNet Hu et al. (2021)	0.34	0.77	0.99	1.46	3.97	0.93	-	-	-
TFA-PFE Li et al. (2023)	1.44	2.50	-	0.76	1.62	-	-	-	-
SINC Speth et al. (2023)	0.61	1.84	0.99	0.59	1.83	0.99	-	-	-
EfficientPhys Liu et al. (2023a)	-	-	-	1.14	1.81	0.99	13.47	21.32	0.21
Contrast-Phys+ Sun and Li (2024)	0.48	0.98	0.99	**0.21**	0.80	0.99	-	-	-
Spore Zhang et al. (2025)	1.05	2.23	0.95	-	-	-	-	-	-
RhythmFormer Zou et al. (2025)	0.29	0.48	0.99	1.03	1.79	0.99	3.07	**6.81**	**0.86**
**Ours**	**0.27**	**0.46**	**0.99**	0.94	1.75	**0.99**	**2.89**	6.89	0.85

#### 4.2.1 PURE and UBFC-rPPG

For PURE, we follow Špetlík et al. (2018); Lu et al. (2021) and use the first 60% of subjects for training and the remaining 40% for testing, with no subject overlap. For UBFC-rPPG, we follow Lu et al. (2021); Song et al. (2021) and use the first 30 subjects for training and the remaining 12 subjects for testing. On PURE, our method achieves the best results across all metrics, reaching MAE =0.27 , RMSE =0.46 , and ρ=0.99 , suggesting the benefit of MDFS’s multi-scale difference cues. On UBFC-rPPG, our method remains highly competitive with MAE =0.94 , RMSE =1.75 , and ρ=0.99 , indicating stable waveform consistency.

#### 4.2.2 MMPD

For MMPD, we perform a sequential split into training, validation, and test sets with a ratio of 7:1:2. Our method achieves the lowest MAE =2.89 , while obtaining RMSE =6.89 and ρ=0.85 , both close to the best-performing baseline, likely aided by SCOPE’s gated refinement under stronger noise. Overall, these intra-dataset results demonstrate the effectiveness and robustness of the proposed method across datasets of varying scale and complexity.

**FIGURE 2:**
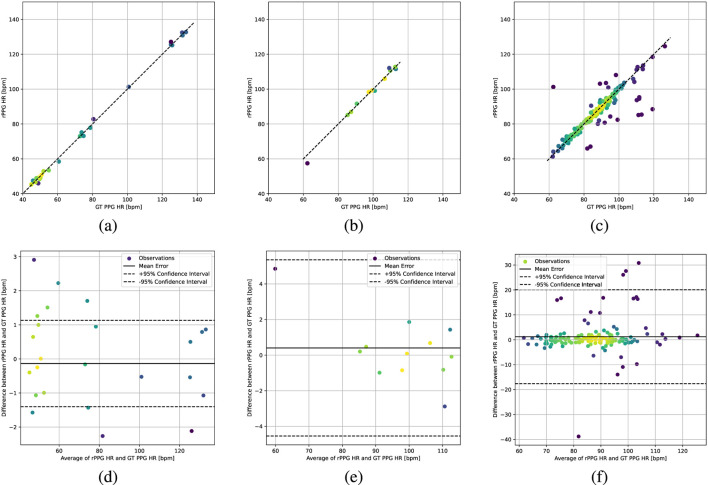
Intra-dataset evaluation results. Top row: scatter plots on **(a)** PURE, **(b)** UBFC-rPPG, and **(c)** MMPD. Bottom row: Bland-Altman plots on **(d)** PURE, **(e)** UBFC-rPPG, and **(f)** MMPD.

We visualize prediction agreement using scatter and Bland–Altman plots on PURE, UBFC-rPPG, and MMPD in Figure 2. Predictions closely match ground truth with near-zero bias and most samples within the 95% limits of agreement, indicating stable HR estimation.

**FIGURE 3:**
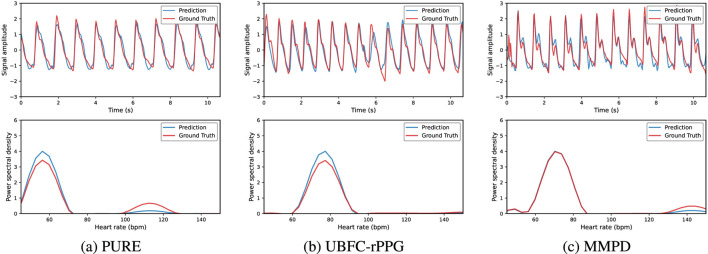
Visualization of rPPG predictions on PURE, UBFC-rPPG, and MMPD datasets. Each column shows time-domain signal comparisons (top) and power spectral density comparisons (bottom), demonstrating waveform consistency and dominant frequency accuracy across datasets. **(a)** PURE. **(b)** UBFC-rPPG. **(c)** MMPD.

To further illustrate the signal reconstruction quality, examples of predicted pulse signals in the time and frequency domains are shown in Figure 3. By comparing the time-domain waveforms and frequency-domain power spectra, the model’s ability to suppress noise and motion artifacts can be intuitively evaluated. The predicted signals exhibit strong agreement with ground truth, accurately capturing the characteristic peaks and variations essential for heart rate estimation.

### 4.3 Cross-dataset evaluation

For cross-dataset evaluation, we follow Liu et al. (2023b) and consider transfer learning settings where models are trained on a source dataset (UBFC-rPPG or PURE) and directly tested on a different target dataset without fine-tuning. We use 80% of the source dataset for training and 20% for validation, and report results on PURE, UBFC-rPPG, and MMPD in Table 2.

**TABLE 2:** Cross-dataset evaluation results on PURE, UBFC-rPPG, and MMPD. The best results are highlighted in **bold**, and the second-best results are underlined.

Method	Train set	Test set														
		PURE					UBFC-rPPG					MMPD				
		MAE ↓	RMSE ↓	MAPE ↓	ρ↑	SNR ↑	MAE ↓	RMSE ↓	MAPE ↓	ρ↑	SNR ↑	MAE ↓	RMSE ↓	MAPE ↓	ρ↑	SNR ↑
GREEN Verkruyet al. (2008)	—	10.09	23.85	10.28	0.34	−2.66	19.73	31.00	18.72	0.37	−11.18	21.73	27.72	24.44	−0.02	−14.34
ICA Poh et al. (2010)	—	4.77	16.07	4.47	0.72	5.24	16.00	25.65	15.35	0.44	−9.91	18.57	24.28	20.85	0.00	−13.84
CHROM De Haan and Jeanne (2013)	—	5.77	14.93	11.52	0.81	4.58	4.06	8.83	3.84	0.89	−2.96	13.63	18.75	15.96	0.08	−11.74
LGI Pilz et al. (2018)	—	4.61	15.38	4.96	0.77	4.50	15.80	28.55	14.70	0.36	−8.15	17.02	23.28	18.92	0.04	−13.15
POS Wang et al. (2016)	—	3.67	11.82	7.25	0.88	6.87	4.08	7.72	3.93	0.92	−2.39	12.34	17.70	14.43	0.17	−11.53
DeepPhys Chen and McDuff (2018)	UBFC	5.54	18.51	5.32	0.66	4.40	—	—	—	—	—	17.50	25.00	19.27	0.05	−11.72
	PURE	—	—	—	—	—	1.21	2.90	1.42	0.99	1.74	16.92	24.61	18.54	0.05	−11.53
PhysNet Yu et al. (2019)	UBFC	8.06	19.71	13.67	0.61	6.68	—	—	—	—	—	10.24	16.54	12.46	0.29	−8.15
	PURE	—	—	—	—	—	0.98	2.48	1.12	0.99	1.09	13.22	19.61	14.73	0.23	−10.59
TS-CAN Liu et al. (2020)	UBFC	3.69	13.80	3.39	0.82	5.26	—	—	—	—	—	14.01	21.04	15.48	0.20	−10.18
	PURE	—	—	—	—	—	1.30	2.87	1.50	0.99	1.49	13.94	21.61	15.14	0.20	−9.94
PhysFormer Yu et al. (2022)	UBFC	12.92	24.36	23.92	0.47	2.16	—	—	—	—	—	12.10	17.79	15.41	0.17	−10.53
	PURE	—	—	—	—	—	1.44	3.77	1.66	0.98	0.18	14.57	20.71	16.73	0.15	−12.15
EfficientPhys Liu et al. (2023a)	UBFC	5.47	17.04	5.40	0.71	4.09	—	—	—	—	—	13.78	22.25	15.15	0.09	−9.13
	PURE	—	—	—	—	—	2.07	6.32	2.10	0.94	−0.12	14.03	21.62	15.32	0.17	−9.95
Spiking-Phys Liu et al. (2025)	UBFC	3.83	—	5.70	0.83	—	—	—	—	—	—	14.15	—	16.22	0.15	—
	PURE	—	—	—	—	—	2.80	—	2.81	0.95	—	14.57	—	16.55	0.14	—
RhythmFormer Zou et al. (2025)	UBFC	0.97	3.36	1.60	0.99	**12.01**	—	—	—	—	—	9.08	**15.07**	11.17	**0.53**	−7.73
	PURE	—	—	—	—	—	0.89	1.83	0.97	0.99	**6.05**	**8.98**	**14.85**	11.11	**0.51**	−8.39
**Ours**	UBFC	**0.80**	**2.90**	**1.33**	**0.99**	11.89	—	—	—	—	—	**8.99**	16.03	**10.25**	0.38	**−5.94**
	PURE	—	—	—	—	—	**0.84**	**1.78**	**0.93**	**0.99**	5.72	9.47	16.50	**10.95**	0.42	**−7.76**

As shown in Table 2, our method consistently achieves strong cross-dataset generalization and outperforms traditional signal-processing baselines (e.g., GREEN Verkruysse et al. (2008) and ICA Poh et al. (2010)) by a large margin. In PURE ↔ UBFC transfer, our method obtains the best results across most metrics, reaching ρ=0.99 with low errors in both directions. More importantly, when transferring to the more challenging MMPD dataset, our method remains competitive and achieves the best or second-best performance on several key metrics, demonstrating robust generalization under large distribution shifts.

**FIGURE 4:**
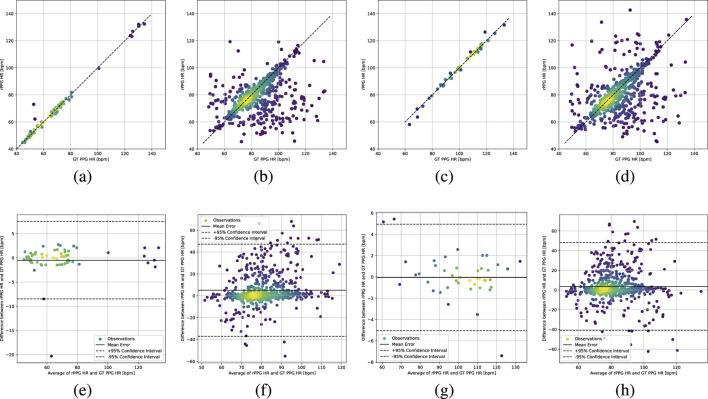
Cross-dataset evaluation results. **(a-d)** Scatter plots: training on UBFC-rPPG and testing on PURE and MMPD **(a,b)**, training on PURE and testing on UBFC-rPPG and MMPD **(c,d)**. **(e–h)** Bland–Altman plots with the same training and testing configurations as **(a–d)**.

Figure 4 presents the scatter plots and Bland–Altman plots for cross-dataset evaluation. When testing on UBFC and PURE, most predicted points cluster near the diagonal with small errors and most points within the 95% confidence interval, indicating good generalization and stable performance. The MMPD dataset shows slightly larger variability due to its more complex scenarios, but the model still achieves acceptable accuracy. Overall, our method demonstrates strong cross-domain generalization across different datasets.

### 4.4 Ablation study

#### 4.4.1 Impact of SCOPE

We conduct ablation experiments to evaluate the contribution of SCOPE under the cross-dataset setting, where the model is trained on UBFC-rPPG or PURE and tested on MMPD. As shown in Table 3, we compare five variants: no-SCOPE removes the entire module; SCOPE-CBAM replaces SCOPE with the standard CBAM module at the same insertion position under identical training settings; SCOPE-ChannelOnly and SCOPE-SpatialOnly retain only one refinement branch; and SCOPE-NoGate disables the gated fusion mechanism. Ours denotes the complete SCOPE design.

**TABLE 3:** Ablation study of SCOPE variants under cross-dataset setting (train on PURE/UBFC-rPPG, test on MMPD). The best results are highlighted in bold.

Method	Train set	MAE ↓	RMSE ↓	MAPE ↓	ρ↑	SNR ↑
No-SCOPE	UBFC	9.16	16.30	10.47	0.39	−6.75
	PURE	9.48	16.60	10.82	0.36	−7.39
SCOPE-CBAM	UBFC	9.89	16.12	11.58	0.37	−7.81
	PURE	**9.33**	**16.17**	11.47	0.37	−7.92
SCOPE-ChannelOnly	UBFC	10.87	17.83	12.16	0.32	−7.59
	PURE	9.76	16.65	10.97	0.36	**−6.58**
SCOPE-SpatialOnly	UBFC	12.58	19.00	14.64	0.15	−9.95
	PURE	11.99	18.49	13.34	0.21	−8.95
SCOPE-NoGate	UBFC	9.18	16.09	10.79	0.38	−6.06
	PURE	9.89	16.92	**10.75**	0.40	−6.47
Ours	UBFC	**8.99**	**16.03**	**10.25**	**0.38**	**−5.94**
	PURE	9.47	16.50	10.95	**0.42**	−7.76

Compared with *SCOPE-CBAM*, the proposed full SCOPE shows clear advantages in the UBFC → MMPD setting, where all five metrics are improved. Specifically, MAE is reduced from 9.89 to 8.99, RMSE from 16.12 to 16.03, and MAPE from 11.58 to 10.25, while the correlation coefficient improves from 0.37 to 0.38 and SNR from −7.81 to −5.94. Beyond the comparison with *SCOPE-CBAM*, the results of the partial variants further support the effectiveness of the complete design. In general, removing either refinement branch or disabling the gated fusion leads to inferior overall performance, indicating that both channel–spatial cooperation and adaptive gated residual fusion are important to the cross-dataset performance of SCOPE.

In the PURE → MMPD setting, the results are more mixed. Although MAE and RMSE are slightly worse than those of *SCOPE-CBAM*, the full SCOPE still achieves better MAPE, higher correlation, and better SNR. We would like to emphasize that this does not mean that SCOPE is ineffective on PURE. On the contrary, as shown later by the intra-dataset ablation results, SCOPE remains superior to *SCOPE-CBAM* when both training and testing are conducted within PURE. Therefore, the weaker gains on MAE and RMSE in the PURE → MMPD transfer should be interpreted as a cross-domain generalization issue rather than a deficiency of SCOPE itself. A more plausible explanation is that PURE, as a source dataset, is relatively limited in scale and diversity, which restricts the coverage of appearance, illumination, motion, and subject variations required for robust transfer to the more challenging MMPD domain. Under such a setting, the task-oriented refinement of SCOPE still improves signal consistency and physiological relevance, as reflected by the better MAPE, correlation, and SNR, while the absolute-error metrics MAE and RMSE remain more sensitive to cross-domain amplitude mismatch and distribution shift. These observations suggest that the advantage of SCOPE is not solely attributable to inserting a generic attention block, but is related to its task-oriented refinement design for rPPG features.

To further investigate this point, we conduct an additional intra-dataset ablation on both UBFC-rPPG and PURE. As shown in Table 4, the full SCOPE consistently outperforms *SCOPE-CBAM* and the other partial variants across nearly all metrics on both datasets under intra-dataset evaluation. In particular, on PURE, the complete SCOPE achieves better MAE, RMSE, MAPE, and SNR than *SCOPE-CBAM*, which directly confirms that SCOPE itself is effective on the PURE domain. This result helps clarify that the mixed behavior in the PURE → MMPD setting does not arise from the failure of the proposed module, but is more likely caused by the limited cross-domain coverage provided by PURE as a source domain. When the train–test domain gap is removed, SCOPE shows a stable advantage over the generic CBAM baseline.

**TABLE 4:** Ablation study of different SCOPE variants on PURE and UBFC-rPPG. The best results are highlighted in bold.

Method	PURE					UBFC-rPPG				
	MAE ↓	RMSE ↓	MAPE ↓	ρ↑	SNR ↑	MAE ↓	RMSE ↓	MAPE ↓	ρ↑	SNR ↑
No-SCOPE	0.36	0.58	0.44	0.99	11.51	1.17	1.83	1.41	0.99	4.50
SCOPE-CBAM	0.29	0.48	0.36	0.99	12.54	0.99	1.85	1.26	0.99	6.11
SCOPE-ChannelOnly	0.33	0.52	0.39	0.99	11.53	0.99	1.89	1.25	0.98	8.33
SCOPE-SpatialOnly	0.33	0.50	0.42	0.99	13.35	0.99	1.66	1.20	0.99	8.47
SCOPE-NoGate	0.31	0.49	0.35	0.99	12.04	0.99	1.79	1.23	0.99	9.03
Ours	**0.27**	**0.46**	**0.34**	**0.99**	**15.24**	**0.94**	**1.74**	**1.18**	**0.99**	**9.51**

**FIGURE 5:**
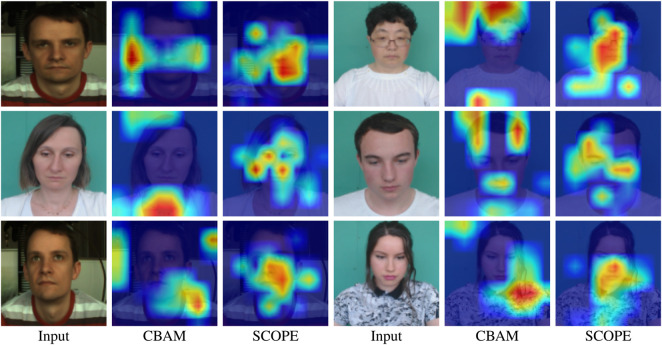
Grad-CAM visualization comparison between CBAM and SCOPE on six example images. The examples are arranged in three rows, with two examples per row. Within each example, the input frame, the Grad-CAM result of CBAM, and the Grad-CAM result of SCOPE are shown from left to right.

To qualitatively examine whether SCOPE guides the network toward more rPPG-relevant facial regions than the generic CBAM design, we further apply Grad-CAM to visualize their activation maps on representative facial frames. As shown in Figure 5, CBAM tends to respond strongly to broader surrounding regions, whereas SCOPE produces more compact activations concentrated on the forehead, cheeks, and central facial areas. These regions are widely regarded as physiologically informative for rPPG estimation. Overall, the Grad-CAM visualizations provide qualitative evidence that SCOPE is better aligned with the characteristics of rPPG signals, rather than functioning merely as a generic feature enhancement module.

#### 4.4.2 Impact of the MDFS

Table 5 compares three variants. Specifically, MDFS-OriginalOnly denotes the baseline that keeps only the original-frame branch without any multi-scale frame-difference branches. MDFS-Fixed introduces the same multi-scale frame-difference branches as MDFS but combines them using fixed, hand-crafted fusion coefficients. Ours denotes the complete framework with MDFS.

**TABLE 5:** Ablation study on cross-dataset evaluation and influence of the MDFS on MMPD.

Method	Train set	MAE ↓	RMSE ↓	MAPE ↓	ρ↑	SNR ↑
MDFS-OriginalOnly	UBFC	32.37	35.96	36.87	−0.006	−22.78
	PURE	12.10	15.75	14.35	−0.009	−12.39
MDFS-fixed	UBFC	9.42	16.69	10.95	0.40	−6.26
	PURE	9.86	17.06	10.82	0.38	−6.57
Ours	UBFC	8.99	16.03	10.25	0.38	−5.94
	PURE	9.47	16.50	10.95	0.42	−7.76

As shown in Table 5, FusionStemOriginalOnly suffers a pronounced performance drop, with correlations close to zero or even negative, indicating that single-scale inputs are insufficient under domain shift. FusionStemFixed markedly reduces the errors; for example, MAE decreases from 32.37 to 9.42 on the UBFC-to-MMPD transfer, which highlights the importance of multi-scale temporal differences for cross-dataset robustness. Overall, the full MDFS achieves the best trade-off across metrics, validating the effectiveness of adaptive multi-scale temporal modeling.

## 5 Discussion

Recent sequence models have improved rPPG estimation by enhancing long-range temporal aggregation. However, under practical low-SNR conditions, rPPG signals are easily dominated by motion and illumination artifacts, and the effectiveness of temporal modeling is often constrained by two factors: (1) raw-frame or single-scale difference inputs provide fragile temporal cues, and (2) generic attention designs lack controllable spatial–channel refinement, which may amplify non-pulsatile responses under domain shift. Therefore, improving rPPG performance is not only dependent on stronger temporal backbones, but also requires explicitly constructing noise-resilient temporal representations and performing rPPG-specific feature enhancement.

From Table 2, the proposed method shows stronger cross-dataset generalization than both classical signal-processing approaches and representative deep models. In particular, the transfer between UBFC-rPPG and PURE remains stable, while transfer to MMPD is more challenging due to larger distribution shift. This observation is consistent with the scatter and Bland–Altman plots in Figure 4, where predictions under UBFC ↔ PURE remain tightly aligned with small bias and most samples fall within the 95% limits of agreement, whereas the point clouds become more dispersed and the agreement intervals widen on MMPD.

To further analyze the sources of robustness, ablation results in Tables 3, 5 verify the effectiveness of the two key designs. As shown in Table 5, removing multi-scale difference cues causes severe degradation under cross-dataset transfer, while introducing multi-scale differences substantially improves errors and correlation, indicating that explicit multi-scale temporal cues are critical for reliable pulse modeling under domain shift. Meanwhile, Table 3 shows that replacing SCOPE with generic attention brings limited gains, and removing the gating mechanism consistently reduces performance, suggesting that controlled spatial–channel refinement with gated residual fusion is essential for suppressing non-pulsatile interference in challenging scenarios.

Although the proposed method achieves consistent improvements, cross-dataset transfer to MMPD still exhibits wider error ranges, indicating that rPPG estimation under complex motion and illumination remains difficult. In future work, we will explore stronger domain-robust learning strategies and more diverse training data to further enhance generalization under extreme acquisition conditions.

## 6 Conclusion

This paper presents a robust rPPG estimation framework designed to address the low-SNR and noise-sensitive nature of remote physiological measurement. The proposed approach integrates a Multi-scale Difference Fusion Stem (MDFS) to construct noise-resilient temporal representations and a Spatio-Channel Operator with Selective Pulse Enhancement (SCOPE) to perform controlled, rPPG-specific feature refinement. Together, these designs enable more reliable extraction of pulse-related information under motion and illumination interference. Extensive experiments on PURE, UBFC-rPPG, and MMPD demonstrate consistent improvements over representative methods in both intra-dataset and cross-dataset evaluations, with particularly strong robustness under domain shift. These results suggest that effective rPPG estimation depends not only on stronger temporal backbones, but also on how temporal cues are constructed and how pulse-relevant features are selectively enhanced. From an application perspective, the proposed framework provides a practical solution for contactless heart-rate monitoring in real-world scenarios such as telemedicine and daily health assessment, where acquisition conditions are often uncontrolled. Future work will further explore domain-aware strategies to improve robustness under more extreme real-world conditions.

## Data Availability

The original contributions presented in the study are included in the article/supplementary material, further inquiries can be directed to the corresponding author.
